# Erythropoietin/erythropoietin receptor system is involved in angiogenesis in human neuroblastoma

**DOI:** 10.1111/j.1365-2559.2007.02653.x

**Published:** 2007-04-01

**Authors:** D Ribatti, P L Poliani, V Longo, D Mangieri, B Nico, A Vacca

**Affiliations:** Department of Human Anatomy and Histology, University of Bari Medical School Bari, Italy; 1 Department of Pathology, University of Brescia Medical School Brescia; 2 Department of Internal Medicine and Clinical Oncology, University of Bari Medical School Bari, Italy

**Keywords:** angiogenesis, erythropoietin, erythropoietin receptor, human neuroblastoma

## Abstract

Ribatti D, Poliani P L, Longo V, Mangieri D, Nico B & Vacca A (2007) *Histopathology***50,** 636–641 **Erythropoietin/erythropoietin receptor system is involved in angiogenesis in human neuroblastoma**

**Aims:**

Previous studies have shown that increased vascularity is associated with tumour progression in human neuroblastoma (NB). The involvement of erythropoietin (Epo) in tumour angiogenesis has also been reported. The aim of this study was to correlate microvascular density and Epo/Epo-receptor (EpoR) expression in endothelial and tumour cells to the clinical stage of NB.

**Methods and results:**

Specimens of NB obtained from 20 patients were investigated immunohistochemically by using anti-CD31, anti-Epo and anti-EpoR antibodies. The extent of angiogenesis was found to be up-regulated in advanced disease. In keeping with this observation, Epo/EpoR expression in tumour and endothelial cells, respectively, was also highly correlated with the extent of angiogenesis and higher clinical stage.

**Conclusions:**

The correlation of Epo/EpoR expression with angiogenesis and tumour progression suggests the presence of a loop in the Epo–EpoR system. Epo is secreted by tumour cells and affects vascular endothelial cells via its receptor, promoting tumour angiogenesis in a paracrine manner. Data suggest that Epo represents an important mediator in NB angiogenesis. Understanding the mechanisms of NB angiogenesis provides the basis for a rational approach to the development of antiangiogenic therapy in patients affected by NB.

## Introduction

Neuroblastoma (NB) is the most common malignant tumour in infants and the fourth most common in children > 1 year old.^1^ Angiogenesis, i.e. the formation of new blood vessels from pre-existing ones, is important in carcinogenesis as well as in the tumour progression of human solid and haematological tumours.^2^, ^3^ NB, in particular, shows wide histological variability that reflects different steps in tumour maturation and angiogenesis appears to play an important role in determining tumour phenotype.^4^ Many angiogenic stimulators, such as vascular endothelial growth factor (VEGF), fibroblast growth factor-2 (FGF-2), as well as inhibitors, such as tissue inhibitors of matrix metalloproteinases (MMPs), have been detected in neuroblastic tumours.^5^–^7^ High tumour vascularity correlates with metastatic disease, *myc* amplification, unfavourable biology and poor outcome; by contrast, low tumour vascularity is associated with favourable prognostic features, such as localized disease and favourable histology.^8^–^11^

Erythropoietin (Epo) is a low-molecular-weight glycoprotein hormonal stimulator of erythropoiesis produced in the fetal liver and subsequently in the adult kidney.^12^ Epo exerts its action through its specific receptor (EpoR), a member of the cytokine receptor superfamily, which is mainly expressed on erythroid colony-forming units.^13^

Despite its original function, Epo is a pleiotropic cytokine that exerts various biological functions in many different non-haematopoietic tissues. Angiogenesis has been identified as one of the extrahaematopoietic functions of Epo.^14^ The role of Epo in angiogenesis has not yet been clarified, although many critical roles for Epo have been reported. EpoR has been found to be expressed on endothelial cells from different tissues.^15^ Moreover, Epo induces endothelial cell proliferation and migration^15^–^17^ and has been shown to stimulate angiogenesis in rat aortic rings *in vitro.*^18^ We have demonstrated that recombinant human Epo (rhEpo) induces a proangiogenic phenotype in human endothelial cells.^19^ This phenotype includes both early (i.e. increase in cell proliferation and MMP-2 production) and late (differentiation into vascular tubes) angiogenic events. Accordingly, after rhEpo exposure, endothelial cells express EpoR that binds to JAK-2 and induces its transient activation. It is interesting to note that JAK-2 is involved in the intracellular signalling of receptors for various angiogenic cytokines,^20^ suggesting a possible role for JAK-2/STAT-5 signalling in cytokine-mediated angiogenesis. In the chick embryo chorioallantoic membrane (CAM) assay, the angiogenic activity of rhEpo is quantitatively and qualitatively similar to that exerted by FGF-2 and colocalization of EpoR and factor VIII-related antigen has been demonstrated on CAM endothelial cells. Jaquet *et al*.^21^ have reported that Epo has a similar angiogenic potential to VEGF in stimulating human adult myocardial endothelial cells.

The potential role of Epo in angiogenesis may be considered as a subsidiary of its possible function in improving overall tissue oxygenation and inhibition of apoptosis. The expression of EpoR in tumour vascular endothelium suggests that Epo may affect the tumour microenvironment, perhaps by stimulating tumour angiogenesis.^14^

Expression of Epo/EpoR in human NB has been reported,^31^ but a morphometric and quantitative study is still lacking. In this study, we correlated Epo/EpoR expression with vascular density across the whole spectrum of neuroblastic tumours reflecting different clinical stages. Our aim was to establish a potential role for Epo in angiogenesis in NB.

## Materials and methods

### Human nb tumour samples

Paraffin-embedded sections of 20 archival primary adrenal and paraspinal human NB tumour samples were obtained by the Department of Pathology of the University of Brescia Medical School. The clinical stages were formulated according to the International Neuroblastoma Staging System (INSS).^22^ INSS stages I and IIa include low-risk small tumours which can be managed by surgery alone; stages IIb and III include patients with intermediate-risk disease characterized by large, unresectable, localized tumours without structural chromosomal defects; stage IV includes high-risk tumours characterized by disseminated disease with gross genetic alterations, such as amplification of *N-myc*; stage IVs includes infants < 1 year old at diagnosis with a small primary tumour and dissemination of disease.

### Immunohistochemistry

A murine monoclonal antibody (MAb) against the human endothelial cell marker CD31, a more sensitive marker for endothelial cells than factor VIII antigen,^23^ (MAb 1A10; DakoCytomation, Glostrup, Denmark), and two rabbit polyclonal antibodies against Epo and EpoR (N19 and C20 Santa Cruz Biotechnology, Santa Cruz, CA, USA) were used. The anti-Epo and anti-EpoR antibodies are two affinity-purified rabbit polyclonal antibodies raised against a peptide mapping at the N-terminus of Epo and, respectively, against amino acids 21-214 mapping near the N-terminus of EpoR of human origin. Briefly, sections were collected on 3-amino-propyl-triethoxysilane-coated slides, deparaffinized by the xylene–ethanol sequence, rehydrated in a graded ethanol scale and in Tris-buffered saline (TBS, pH 7.6) and incubated overnight at 4°C with the MAb 1A10 (1 : 25 in TBS) and the polyclonal antibodies N19 and C20 (1 : 200 in TBS), after prior antigen retrieval by heating the sections in a pressure cooker in 1 mmol/l ethylenediamine tetraacetic acid buffer, pH 8.0, for 1.5 h. As for CD31 immunohistochemistry, sections were incubated with biotinylated IgG and then with peroxidase-conjugated streptavidin (LSAB2; DakoCytomation). Immunodetection of CD31 was by diaminobenzidine, while Epo and EpoR detection was performed with alkaline phosphatase–antialkaline phosphatase (DakoCytomation) and fast red as chromogen, followed by haematoxylin counterstaining. Negative controls included an unrelated monoclonal IgG1 produced by the P3X63/Ag8 mouse secretory myeloma replacing the antibody, for the MAb against CD31^24^ and preincubation with a 10-fold excess of specific blocking peptide (Santa Cruz) for the polyclonal antibodies against Epo and EpoR.

### Microvessel density, Epo and EpoR expression counting

Four to six 200× fields for each slide covering almost the whole of three sections per sample were examined with a 144-intersection point square reticulum (0.78 mm^2^) inserted in the eyepiece. Particular attention was paid to selection of microvessels, i.e. capillaries and small venules, from all CD31+ vessels. They were identified as transversely sectioned tubes with a single layer of endothelial cells, with or without a thin basement membrane. Each assessment was agreed upon in turn. Microvessels were counted with a planimetric point-count method with slight modifications to restrict counting to transversely cut microvessels occupying the reticulum intersection points.^25^ As the microvessel diameter was smaller than the distance between adjacent points, only one transversely sectioned microvessel could occupy a given point. Microvessels transversely sectioned outside the points and those longitudinally or tangentially sectioned were omitted. Therefore, it was sufficiently certain that a given microvessel was counted only once, even in the presence of several of its section planes. As almost the entire section was analysed per sample and as transversally sectioned microvessels hit the intersection points randomly, the method allowed objective counts. Tumour and endothelial cells positive for CD31, Epo and EpoR were counted in four to six fields covering the whole of each of three sections adjacent to those stained for microvessels and means ± 1 SD and medians were determined for each section, sample and group of samples. All analyses were simultaneously assessed without knowledge of the final pathological diagnosis by two investigators with a double-headed light microscope (Axioplan II; Zeiss, Oberkochen, Germany). The relationship between microvessel density, Epo and EpoR expression and clinical stage was examined by χ^2^ test or logistic regression analysis. Statistical significance was defined as *P* < 0.05.

## Results

**Figure 1:**
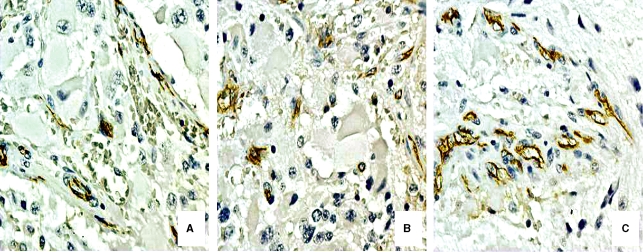
Immunohistochemistry for CD31 in human neuroblastoma. The microvessel density is higher in stage IVs neuroblastoma (**C**) compared with stage III (**B**) and I (**A**)

Histologically, in all the samples examined, microvessels have been defined as endothelial cells clustered in nests or tubes, transversely or longitudinally sectioned, with or without a small lumen. Figure 1 shows representative sections of low and high vascularity of NB immunohistochemically stained with an anti-CD31 antibody. The microvessel density in stage IVs (Figure 1C) was higher compared with stages III (Figure 1B) and I (Figure 1A).

**Figure 2:**
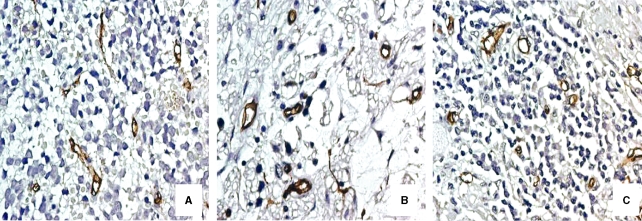
Immunohistochemistry for erythropoietin receptor (EpoR) in human neuroblastoma. The immunoreactivity of endothelial cells for EpoR is higher in stage IVs neuroblastoma (**C**) compared with stage III (**B**) and I (**A**)

**Figure 3:**
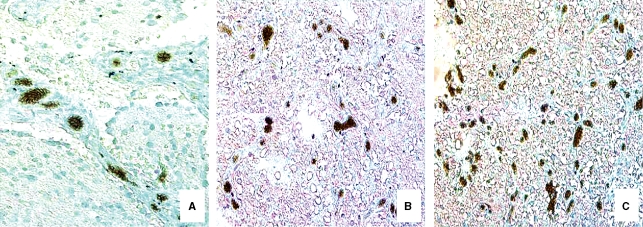
Immunohistochemistry for erythropoietin (Epo) in human neuroblastoma. Few tumour cells are immunoreactive for Epo in stages I (**A**) and III (**B**), while immunoreactivity is greatly increased in stage IVs (**C**)

EpoR was diffusely and strongly expressed on endothelial cells within neoplastic nodules (Figure 2), whereas in most cases Epo showed a weak and focal cytoplasmic granular pattern in a few NB cells in stages I and III (Figure 3A,B), while this immunoreactivity increased in stage IVs (Figure 3C).

Table 1 shows the correlation between microvessel, Epo and EpoR counts and histological tumour grade. The comparison of microvessel counts between histological groups revealed statistically significant differences. When differences were sought between groups, significantly higher counts were found in stage IVs compared with other stages (*P <* 0.001). Regarding differences in microvessel, Epo and EpoR expression in both endothelial and tumour cells, within-group comparison showed that both counts were always significantly correlated. There was a trend for these three parameters to increase with INSS stage. The counts of patients with stage IVs NB were significantly higher (*P <* 0.001) than those of patients with III + IV and, respectively, I + II NB.

**Table 1:** Correlation between International Neuroblastoma Staging System (INSS) stage, microvessel density, erythropoietin (Epo) expression in tumour cells and erythropoietin receptor (EpoR) expression in endothelial cells, expressed as means ± SD of the number of positive cells

INSS stages	Cases	Microvessel density	Epo expression, tumour cells	EpoR expression, endothelial cells
I + II	10	10 ± 5	9 ± 2	5 ± 2
III + IV	5	15 ± 4*	14 ± 3*	8 ± 4*
IVs	5	30 ± 5*	25 ± 4*	25 ± 6*

* *P <* 0.001 compared with I + II.

## Discussion

Angiogenesis and tumour progression in human NB have been correlated. Our data demonstrate that the extent of angiogenesis in human NB, evaluated as microvessel density, increases progressively along with clinical stage. Different molecules and angiogenic factors are involved in tumour angiogenesis in human NB. We have demonstrated that the Epo/EpoR system is involved in NB angiogenesis and that expression of Epo in tumour cells and of EpoR in endothelial cells increases in parallel with grade of malignancy and is highly correlated with the extent of angiogenesis.

Tumour angiogenesis does not depend on a single molecule, since many angiogenic inducers and inhibitors are expressed simultaneously. The presence of an autocrine-paracrine Epo–EpoR system in tumours and the possible effects of Epo on tumour microenvironment and angiogenesis are consistent with a complex biology for Epo–EpoR signalling in cancer.

Kayser and Gabius^26^ first suggested that human tumours express EpoR. In their study, 81% of human lung carcinoma tissues possessed Epo-binding sites, detected by the use of biotinylated rhEpo. EpoR transcripts and EpoR protein were subsequently demonstrated in human renal carcinoma,^27^ tumours of the cervix and other organs of the female reproductive tract^28^–^30^ and in various specimens of common paediatric tumours, including neuroblastomas, brain tumours, hepatoblastomas and Wilms' tumours.^31^ By immunohistochemistry, EpoR has been shown to be expressed in breast carcinoma^32^–^34^ and in vestibular schwannoma.^35^ Yasuda *et al*.^36^ have reported the expression of Epo in several malignant human cell lines, suggesting that they express Epo and EpoR regardless of origin, type, genetic characteristics and biological properties. Moreover, the authors have shown that tumour cells secrete a very small amount of Epo individually and that most of them respond to hypoxic stimuli by enhanced secretion of Epo.

Batra *et al*.^31^ have demonstrated that most of the common paediatric tumours, including NB, express Epo and EpoR, which promote cell survival genes and increase release of angiogenic growth factors from tumour cells. These findings suggest the presence of a loop in the Epo–EpoR system, i.e. Epo is secreted by NB tumour cells and affects vascular endothelial cells via its receptors and promotes angiogenesis in a paracrine manner. Thus, it is suggested that Epo is an important factor in the angiogenesis of human NB.

Understanding the mechanisms of NB angiogenesis provides a basis for a rational approach to the development of antiangiogenic therapy in patients with NB. Inhibition of Epo signalling, by the injection of anti-Epo MAb or a soluble form of EpoR, resulted in delay in the growth of ovarian and uterine cancers.^28^ In nude mice, Yasuda *et al*.^36^ blocked Epo signalling in xenografts of two representative cell lines by intraperitoneal injection of an EpoR antagonist and found inhibition of angiogenesis and survival of tumour cells, leading to destruction of tumour masses. Our results may have implications for the treatment of human NB by inhibition of the Epo–EpoR system.

## Abbreviations

CAM: chorioallantoic membrane
Epo: erythropoietin
FGF-2: fibroblast growth factor-2
INSS: International Neuroblastoma Staging System
MMP: matrix metalloproteinase
NB: neuroblastoma
rhEpo: recombinant human Epo
TBS: Tris-buffered saline
VEGF: vascular endothelial growth factor
